# Quantification of the CBD-FITC conjugates surface coating on cellulose fibres

**DOI:** 10.1186/1472-6750-8-1

**Published:** 2008-01-09

**Authors:** Ricardo Pinto, António L Amaral, Eugénio C Ferreira, Manuel Mota, Manuel Vilanova, Katia Ruel, Miguel Gama

**Affiliations:** 1 IBB-Institute for Biotechnology and Bioengineering, Centre of Biological Engineering, Universidade do Minho, Campus de Gualtar, 4710-057 Braga, Portugal; 2 Departamento de Tecnologia Química, ESTIG, IPB, Apartado 1038, 5301-854 Bragança, Portugal; 3 ICBAS – Instituto de Ciências Biomédicas de Abel Salazar, Largo do Professor Abel Salazar 2, 4099-003 Porto, Portugal; 4 IBMC – Instituto de Biologia Molecular e Celular, Rua do Campo Alegre, 823, 4150-180 Porto, Portugal; 5 Centre de Recherches sur les Macromolécules Végétales (CERMAV)-UPR CNRS 5301, BP 53, 38041 Grenoble cedex 9, France

## Abstract

**Background:**

Cellulose Binding Domains (CBD) were conjugated with fluorescein isothiocyanate (FITC). The surface concentration of the Binding Domains adsorbed on cellulose fibres was determined by fluorescence image analysis.

**Results:**

For a CBD-FITC concentration of 60 mg/L, a coating fraction of 78% and 110% was estimated for *Portucel *and Whatman fibres, respectively. For a saturating CBD concentration, using Whatman CF11 fibres, a surface concentration of 25.2 × 10^-13 ^mol/mm^2 ^was estimated, the equivalent to 4 protein monolayers. This result does not imply the existence of several adsorbed protein layers.

**Conclusion:**

It was verified that CBDs were able to penetrate the fibres, according to confocal microscopy and TEM-immunolabelling analysis. The surface concentration of adsorbed CBDs was greater on amorphous fibres (phosphoric acid swollen) than on more crystalline ones (Whatman CF11 and Sigmacell 20).

## Background

Due to its high sensitivity and specificity, fluorescence analysis has been extensively used in microarray technology [1], gene expression monitoring [2], protein diffusion [3] or *in vivo *chemical elements uptake and localization studies [4]. Another area to benefit from fluorescence based techniques is protein quantification. The use of either non-covalent [5] or covalent labelling [6,7] increased the detection sensitivity to as low as 40 ng/mL.

Cellulose-Binding Domains (CBD) are modules present in most celulases, being responsible for their high affinity to cellulose crystalline surfaces [8,9]. The CBD used in this work, produced by limited proteolysis, belongs to cellobiohydrolase I (CBHI) of *Trichoderma reesei*, as shown in a previous work [10]. Three tyrosine residues define a flat surface, which may be responsible for the affinity to cellulose [11]. This protein has a single amine, the N-terminal of the linker region, which allows a specific reaction with fluorescein isothiocyanate (FITC). The conjugation with FITC does not affect the CBD interaction with cellulose, since the N-terminal is isolated from the cellulose interacting part of the protein. Indeed, the conjugation of FITC does not modify the CBD adsorption isotherms [12,13]. Since there is only one amine group present in the CBD, the stoichiometry of the conjugation reaction is 1:1. The FITC fluorophore has been attached to antibodies [14,15], to microparticles [16] or to other binding domains [12,17]. Several recombinant CBDs, fused to different proteins, have been produced, as recently reviewed by Shoseyov *et al.*[18].

Cellulose-Binding Domains (CBD) have been used to target functional molecules to cellulose-containing materials [19], to improve pulp properties [20] or as an additive for paper recycling [21]. Bearing in mind that these applications are related to surface effects, in this work we attempted to quantify the CBD surface coverage of cellulose fibres, using the approach based on the use of CBD-FITC previously developed. Our aim was to quantify the protein adsorbed on cellulose fibres and, more specifically, the surface concentration of CBD. This value could, alternatively, be estimated by measuring the specific surface area, by means of the BET isotherm [22]. However, the BET approach is not ideal for porous materials [23]. The presence of CBDs in the interior of the fibres was also investigated.

## Results and Discussion

**Figure 1:**
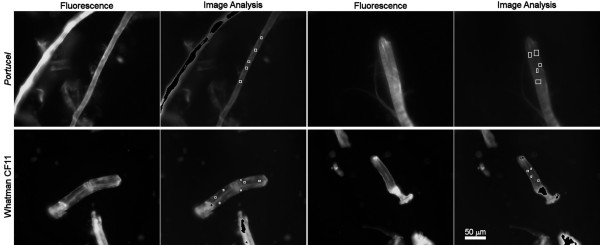
***Portucel *and Whatman CF11 fibres treated with CBD-FITC**. The fibres treated with a concentration of 60 μg/mL (or 2 mg_CBD_/g_fibres_). The images were acquired with an exposure time of 600 ms. The white squares identify the areas selected for analysis. The more fluorescent parts (black regions on the analyzed images) are out of range (calibration shown elsewhere), and were therefore excluded from the analysis.

**Figure 2:**
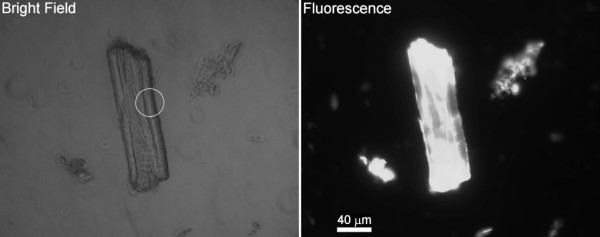
**Whatman CF11 images**. The characteristic curled structure of the fibres (circle).

**Figure 3:**
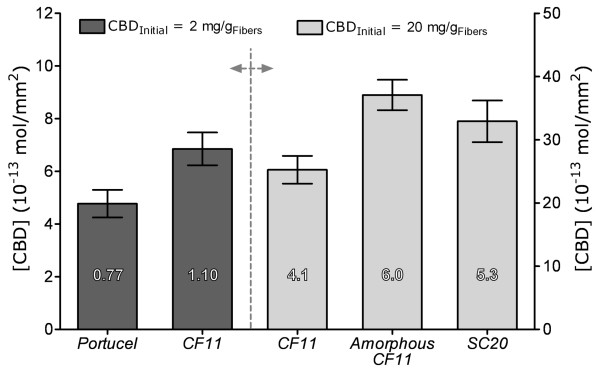
**Estimated surface concentration of adsorbed CBD**. The estimated fraction of surface coverage is indicated in the figure bars. The values shown are based on the assumption that the protein is adsorbed on the external surface of the fibres only. The values are shown with 95% confidence intervals error bars.

In a previous work [21], we have shown that CBDs affect the technical properties of paper fibres (secondary fibres from the paper mill *Portucel*). The concentration of CBDs, used in those experiments, was in the range of 1–2 mg of CBD per gram of fibres. It is arguable whether this relatively low amount of protein is sufficient to cause modifications in the fibres' interfacial properties. This would probably imply a substantial coating of the fibres by CBDs. In this work, we analyzed fibres from *Portucel *treated with CBDs conjugated to FITC, and attempted to estimate the percentage of surface coverage. Fibres treated with only CBDs didn't present any fluorescence. As may be observed in Figure 1, the fibres do not display a uniform distribution of fluorescence. This may be due to the chemical heterogeneity (lignin/hemicellulose) and/or to the variable crystallinity [24]. The regions with less intense fluorescence in the picture were selected for CBD-FITC quantification, since these regions are expected to be more crystalline (Pinto *et al.*, unpublished). The detected fluorescence is produced by CBDs adsorbed on both sides of the fibres (top and bottom). Indeed, the fluorescent radiation crosses the fibres with just a slight reduction in intensity [25]. Figure 2 shows a Whatman CF11 fibre, both on bright field and fluorescence microscopy. As it can be observed in the circled area, this cellulose has a rather smooth surface. The extremities of the fibres are expected to have a higher number of fissures and loosen microfibrils, increasing the available area, and consequently the adsorbing sites for CBDs [12], as indicated by the higher fluorescence emission (Fig. 2). The adsorption of CBDs on Whatman CF11 fibres was analyzed, using a protein concentration of 2 mg/g_fibres_. The estimated surface concentrations of CBDs adsorbed on Portucel and Whatman CF11 fibres (Fig. 1) are shown in Figure 3.

**Figure 4:**
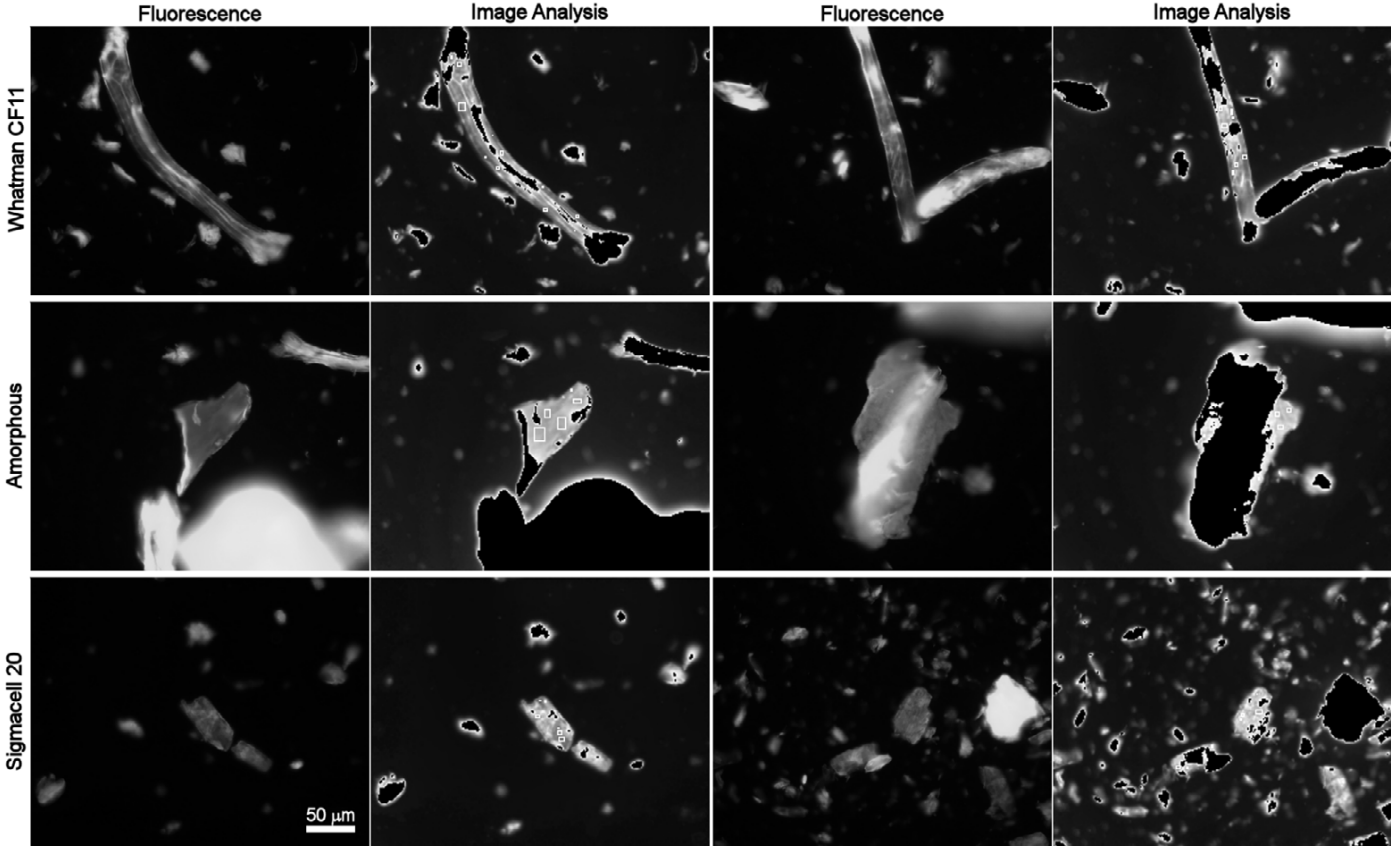
**Images obtained by fluorescence microscopy of Whatman CF11, amorphous and Sigmacell 20 fibres**. The images were obtained before – 1st and 3rd column – and after – 2nd and 4th column – the image analysis of fibres treated with a CBD-FITC concentration of 400 μg/mL. The black areas on the treated images correspond to the fluorescence emission, which is higher than the one used in the calibration. Fluorescence images acquired for 100 ms (CF11) and 80 ms (Amorphous and Sigmacell).

In another experiment, Whatman CF11, amorphous cellulose and Sigmacell 20 fibres were allowed to adsorb CBDs from a much more concentrated CBD solution (400 μg/mL), corresponding to 20 mg/g_Fibres _(Fig. 4). This concentration is expected to saturate the fibres according to the adsorption isotherm.

**Figure 5:**
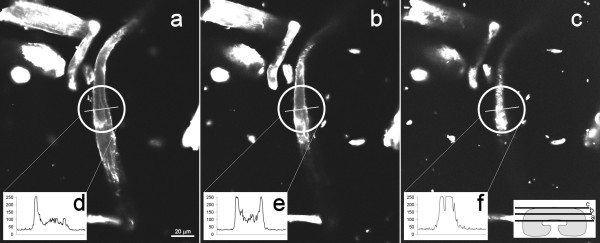
**Images from confocal microscopy**. Three views of a CF11 fibre are shown, as schematized in the right insertion of figure *c*. The insertions *d*, *e *and *f *correspond to the pixels intensity (256 grey levels) obtained at the position indicated by the line (white circle), at different depths. The adsorption conditions were 20 mg_CBD_/g_Fibre_, for 30 minutes of contact. Each image corresponds to an acquisition thickness of 1 μm.

**Figure 6:**
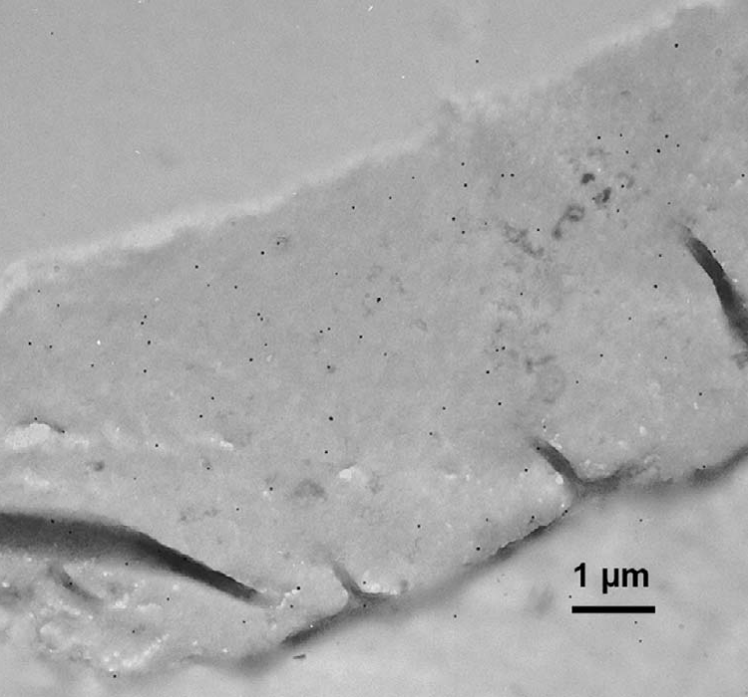
Electron microscopy image of immunolabelling of CBD-treated Whatman CF11 fibres.

Considering the size [26] of a cellobiohydrolase I CBD (3.0 × 1.8 nm), the density of a CBD monolayer corresponds to 3.08 × 10^-13 ^mol/mm^2^. It is also considered that the fluorescent signal is produced by CBDs adsorbed on the two sides of the fibres' external surface. Indeed, the accessible area may be much larger than the one corresponding to a flat, impenetrable fibre. Therefore, an estimate of about 4 layers of CBDs adsorbed in the Whatman CF11 surface results from this reasoning, when the larger concentration of CBDs is used; for a lower CBD concentration, a surface coverage of 77% and 110% is estimated, respectively, for Portucel and Whatman fibres (Fig. 3), dividing the calculated surface concentration by the theoretical monolayer of CBDs. This is much higher than the expected maximum of one layer of adsorbed CBD, at saturation. Although the surface is apparently smooth, the fibres may have irregularities, such as microfissures or holes created by CBDs [27]. The presence of more or less loose microfibrils, large pores or fissures may substantially increase the surface area and the amount of bound proteins, thus leading to a higher fluorescence emission than the theorized monolayer. Indeed, confocal microscopy reveals that the surface has many irregularities (Fig. 5-c), while the inner region presents a homogeneous structure (Fig. 5-a). Another important observation provided by confocal microscopy was that fluorescence in the inner core of the fibres was always superior to the background intensity (Fig. 5-d and Fig. 5-e), indicating that CBDs may have penetrated into the fibre. As a matter of fact, as shown in Figure 5-d, fluorescent material (CBD-FITC) was detected at all depths of the fibre. This observation is supported by immunolabelling of CBD-treated CF11 fibres (Fig. 6). This analysis revealed the presence of CBDs (black spots) in the interior of the fibre. Considering this, we may now explain that the surface concentration of CBDs in Figure 3 arises from the CBDs penetration deep inside the fibres. Another aspect to take into account is that CBDs may not adsorb as a single and well ordered monolayer, but rather as agglomerates [28], thereby increasing the average fluorescence per unit area. Nevertheless, it is quite probable that the CBD coating of the external surface is rather significant.

Amorphous cellulose was prepared, by treating Whatman CF11 with phosphoric acid, which induces the swelling of fibres. As it can be seen in Figure 4, the fibres are larger than the original ones (amorphous Whatman *versus *Whatman CF11). This swelling effect is due to the disruption or loosening of the microfibrils, thus increasing the volume occupied by the total fibre. As a result, the total surface area available for CBD adsorption increases. Consequently, it is expected that the measured CBD fluorescence would be higher than the one obtained for CF11 fibres, mostly due to an easier penetration of CBD into the fibres structure. This was confirmed, as can be seen in Figure 3. The surface coverage increases about 50% (4 versus 6 layers, respectively for Whatman CF11 and amorphous cellulose). This result can arise either from increased CBD affinity for the more amorphous fibres, or to easier penetration, and hence higher concentration in the fibres. Due to the higher fluorescence obtained with these fibres, the majority of the image exceeded the maximum concentration used in the calibration and it had to be excluded in the image analysis (Fig. 4).

Sigmacell 20 is obtained by the separation of crushed cellulose fibres, with an average size of 20 μm. This mechanical treatment expectedly leads to broken ends or loosen fibrils (amorphous material). Then, the adsorption of CBD is expected to be higher than with CF11 and comparable to the amorphous cellulose (see Figure 3). Again, the fibres present a high fluorescence emission corresponding to a high amount of adsorbed CBD: about 5 layers.

## Conclusion

In this work, the surface concentration of CBD adsorbed on cellulose fibres was estimated. The coating values obtained were higher than expected, corresponding in theory to several layers (4 to 6) of CBDs adsorbed on the external surface. However, it has been demonstrated that the CBDs penetrate the fibres. An important amount of CBDs was detected inside the fibres by immunolabelling and confocal microscopy. It seems that a large fraction of the adsorbed CBDs actually penetrate the fibres. The surface coverage values are, undoubtedly, high enough to justify a change in the fibres' surface properties. CBDs may therefore be used as powerful tools to modulate the fibres' surface properties.

## Methods

### Chemicals

Fluorescein isothiocyanate (FITC), SigmaCell Type 20 and Whatman CF11 were obtained from Sigma. Secondary fibres where kindly supplied by *Portucel Viana*. All chemicals were of the highest purity available in the market.

### Amorphous Cellulose Preparation

Amorphous cellulose was prepared by treating Whatman CF11 fibres with phosphoric acid. Briefly, 0.17 g of Whatman CF11 were slowly mixed with 10 mL of cold (4°C) phosphoric acid (85%), and left in contact for 5 minutes. Then, 600 mL of cold water were added and the suspension was filtered through a test sieve (mesh width 71 μm, according to DIN 4188). Finally, the fibres were extensively washed first in tap water and afterwards with distilled water. The obtained material was lyophilized and stored.

### CBD Production

The CBDs were prepared according to the following methodology: the Celluclast^® ^commercial enzymatic preparation (Novozymes A/S, Denmark) was digested with Papain (1:1200, protein basis). The CBDs were separated by ultrafiltration through a 10 kDa membrane (Pellicon 2 TFF System from Millipore, USA) and concentrated by precipitation with ammonium sulphate (Merck, Darmstadt, Germany). After dialysis, the protein was injected on a Sepharose Fast-Flow gel (Amersham Pharmacia Biotech AB, Sweden), and the non-adsorbed protein was collected and lyophilized. The purity and identity (CBD from *T. reesei *CBH I) of this protein has been demonstrated by N-terminal sequencing and MALDI-TOF [10].

### CBD-FITC production

The conjugation of CBD with the labelling probe was carried out by mixing 20 μg of FITC per mg of CBD (2 mg protein/mL in 0.1 M HEPES buffer, pH 9.0). This solution was incubated overnight in the dark, at room temperature, with magnetic stirring. To eliminate the unbound FITC, the labelled CBD mixture was filtered through a BIO-GEL P-4 (BIO-RAD, Hercules, USA) column, previously equilibrated with 50 mM sodium acetate buffer (Panreac, Barcelona, Spain).

### CBD-FITC Adsorption

Adsorption assays of FITC-labelled CBD were carried out at 4°C. The conjugates were allowed to adsorb on cellulose fibres (20 g/L, in 50 mM sodium acetate buffer, pH 5.0), with continuous magnetic stirring, in the dark, for 2 hours. The supernatant with unbound CBD was removed by centrifugation at 3219 RCF for 10 minutes (Heraeus Megafuge 1.0R). The fibres were washed with acetate buffer to remove the non-adsorbed CBD-FITC.

### Image Acquisition

Fluorescence microscopy observations were performed in a Zeiss Axioskop microscope (Zeiss, Oberkochen, Germany) equipped with a Zeiss AxioCam HRc attached camera (Zeiss, Oberkochen, Germany) and using the AxioVision 3.1 software (Zeiss, Oberkochen, Germany). All images were acquired at 1300 × 1030 pixels and 24 bits colour depths (8 bits per channel). The FITC-CBD quantification was performed as described elsewhere [25].

Confocal observation was performed in an Olympus (Tokyo, Japan) Fluoview 1000 in Laser Scanning mode equipped with a 60× UPLSAPO lens, with a numerical aperture of 1.35 and a pinhole size of 105 μm.

### Antiserum preparation

CBD-specific antibodies were produced in a rabbit (*Oryctolagus cuniculus*) maintained under standard conditions of housing with unrestricted access to food and water; these conditions followed European Union Directive no. 86/609/CEE. Briefly, the rabbit was immunized intradermically (i.d.) with a 1:1 suspension of Phosphate Buffered saline (PBS)/Complete Freund's adjuvant containing 500 μg CBD and boosted two weeks later i.d. with a 1:1 suspension of PBS/Incomplete Freund's adjuvant containing 500 μg CBD. Blood was collected three weeks after the second immunization for the preparation of immune serum. Purification of IgG antibodies from this serum sample was performed as follows: the serum sample was equilibrated in a binding buffer (20 mM sodium phosphate, pH 7.0) by overnight dialysis and 3 ml of this preparation was applied to a Protein G HP affinity column (HiTrap, Amersham Biosciences, UK). Bound antibodies were eluted with Glycine -HCl buffer, pH 2.7 and recovered in 50 μl of 1 M Tris-HCl pH 9.0 per ml of eluent, according to the manufacturer's instructions. Recovered IgG antibodies were further equilibrated in PBS in a VIVAPORE concentrator with a 7.5 kDa cutoff membrane (Vivascience, Hanover, Germany) and stored at -80°C in frozen aliquots. The anti-CBD antibody titre of this preparation was determined by ELISA. Specific anti-Sap2 or anti CaS antibodies in mice sera collected by retrorbital bleeding were quantified by ELISA. Polystyrene microtitre plates (Nunc, Roskilde, Denmark) were coated with 20 μg/ml of CBD and incubated o.n. at 4°C. Wells were then saturated for 1 h at room temperature with 1% BSA in PBS. Serial dilutions of the serum samples were then plated and incubated for 2 h at room temperature. After washing, bound antibodies were detected by adding alkaline phosphatase-coupled monoclonal goat anti-rabbit-IgG antibody (Southern Biotechnology Associates, Birmingham, ALA, USA) for 30 min at room temperature. Substrate solution containing p-nitrophenyl phosphate (Sigma, St. Louis, USA) was then added after washing and the reaction was stopped by adding 0.1 M EDTA pH 8.0. The absorbance was measured at 405 nm. The ELISA antibody titres were expressed as the reciprocal of the highest dilution giving an absorbance of 0.1 above that of the control (no serum added). The titre of anti CBD antibodies in the purified IgG preparation was of 4014. No antibodies with this specificity were detected in the control sera from non-immunized rabbits.

### Immunolabelling in Transmission Electron Microscopy

The CBD-treated Whatman CF11 fibres were fixed in a freshly prepared mixture of 0.2% glutaraldehyde (v/v), 2% paraformaldehyde (w/v) in 0.05 M phosphate buffer (pH 7–7.2). Successive periods of vacuum (5 to 10 min) and air inlet were carried out, up to two hours. Afterwards, the fibres were washed 3 × 10 minutes with 0.05 M phosphate buffer. The samples were then dehydrated through a graded series of ethanol and embedded in London Resin White (hard mixture) polymerized for 24 h at 50°C.

Immunolabelling was done on ultrathin transverse sections (500 Å) floating on plastic rings [29]. The sections were floated on several dilutions of the antiserum in 10 mM Tris buffered saline (500 mM NaCl) to determine the optimal ratio of labelling and background [30]. The secondary marker was Protein A-gold (pA G5, BioCell). The gold particles were further enhanced using a silver enhancing Amersham kit. Finally, the thin-sections were transferred on copper-grids, post-stained with 2.5% aqueous uranyl acetate and examined with a Philips CM 200 Cryo-TEM at an accelerating voltage of 80 kV.

To guarantee semi-quantitative comparative labelling, experiments were carried out in parallel on treated and non-treated samples of CBD. Therefore, the exposure to the antibody was identical.

## Authors' contributions

RP carried out the amorphous cellulose, CBD and CBD-FITC production, made the adsorption assays and the image acquisition, participated in the conception of this work and manuscript writing. ALA and ECF helped conceiving the image analysis system. MV produced the antiserum. KR was responsible for the TEM-immunolabeling experiments. MM is the director of the research unit and helped to draft the manuscript. MG participated in the conception and supervision of the work and contributed to the manuscript preparation. All authors read and approved the final manuscript.
